# The Parasitic Eyeworm *Oxyspirura petrowi* as a Possible Cause of Decline in the Threatened Lesser Prairie-Chicken (*Tympanuchus pallidicinctus*)

**DOI:** 10.1371/journal.pone.0108244

**Published:** 2014-09-24

**Authors:** Nicholas R. Dunham, Steven T. Peper, Catherine E. Baxter, Ronald J. Kendall

**Affiliations:** The Wildlife Toxicology Laboratory, The Institute of Environmental and Human Health, Texas Tech University, Lubbock, Texas, United States of America; Universidad de Granada, Spain

## Abstract

Lesser prairie-chickens (*Tympanuchus pallidicinctus*) have been declining range wide since the early 1900's despite efforts to establish conservation and improve their habitat. In early 2014, the lesser prairie-chicken was listed as a threatened species under the U.S Endangered Species Act and the need to find out why they are declining is more important than ever. Nine hunter shot lesser prairie-chickens were donated and sampled for the presence or absence of the eyeworm *Oxyspirura petrowi*, a known parasite that can cause damage to the eye of its host, and common environmental contaminants. Eyeworm infection was found in 7 of 9 birds (78% infection rate) with an infection range between 0–16 *O. petrowi* per bird. Breast, liver, and fat tissue samples from the lesser prairie-chickens were analyzed for the frequency of 20 organochlorine pesticides. Femurs and livers were also tested on these birds for metal contaminants. Pesticides were found in several samples above the detection limits but were still in the low ng/g range. Notable was the ubiquitous presence of endrin aldehyde across all tissues. One femur showed 5.66 µg/g of lead (Pb) but this is still relatively low. No liver samples had elevated mercury (Hg) above detection limits. The presence of these organochlorines is consistent with the historic use of pesticides in this region. With pesticide and metals found in such low levels and parasitic nematode infections at rather high levels, it is recommended that these parasites be further evaluated as a contributing factor to the decline of the lesser prairie-chicken.

## Introduction

Historically, lesser prairie-chickens (*Tympanuchus pallidicinctus*) have thrived throughout much of the southern United States but since the early 1900's their population and range have been diminished by over 90% [1]. Arguably much of their decline has been blamed on anthropogenic factors including habitat loss due to agriculture or habitat fragmentation [2]. In early 2014, the lesser prairie-chicken was placed as a threatened species under the U.S Endangered Species Act which means if proper conservation and management isn't established the lesser prairie-chicken will become an endangered species in the foreseeable future under the law [3]. Recent reports of lesser prairie-chickens flying into stationary objects and other anecdotal reports of these birds flying into objects, as large as vehicles and barns, have led us to wonder if these birds have vision problems or other neurological problems. These problems are often caused through either parasitic infections, contamination by organochlorine pesticides, or metal toxicity which will be examined in this manuscript. With their recent listing as threatened under the Endangered Species Act, the need to find out why they are declining and increased conservation measures are needed now more than ever.

*Oxyspirura petrowi* has been receiving increased attention due to its potential role in negatively impacting gamebirds [4]. This parasite, known as the eyeworm, is a nematode that lives on the surface of and/or behind the eyeball in the lacrimal duct and its associated glands. Eyeworm sizes can range from microscopic egg to well over 15 mm in length [5]. While the lifecycle is not completely known, it has been suggested that the lifecycle of *O. petrowi* is likely similar to *Oxyspirura mansoni* which is known to infect poultry [6]. The lifecycle of *O. mansoni* starts when a gravid female deposits embryonated eggs in the eyes of the host, which are then washed down the naso-lacrimal ducts to the mouth, swallowed, and finally excreted into the feces where they are then ingested by an intermediate host [7]. Research is still underway to determine which arthropods are intermediate hosts for the eyeworm.

Research by Dunham et al. (2014a) on northern bobwhites (*Colinus virginianus*) revealed damage to tissues behind the eyeball and in the nasal-lacrimal glands causing localized hemorrhaging and swelling by these blood-feeding parasites. Increased eyeworm infections could cause severe hemorrhaging and swelling behind the eye, which applies pressure to the optic nerve, probably compromising the bird's vision. Visual impairments could impact their ability to forage, fly efficiently when escaping predators, and reproduce successfully, all of which could ultimately decrease their survivability in the wild.

The eyeworm *O. petrowi* has been previously found in lesser prairie-chickens in Texas [8] and Kansas [1]. A related species of eyeworm (*O. lumsdeni*) has been documented in lesser prairie-chickens in Oklahoma [9]. However, little research has been conducted on the impact that eyeworms could have on the decline of the lesser prairie-chicken.

Most pesticides of the organochlorine class have been banned or restricted for years; however, they are often still very persistent in soil and can be harmful to birds when they are exposed [10]. Organochlorines in avian species can lead to toxic effects such as lethargy, convulsions, and emaciation with more commonly known effects like eggshell thinning and reproductive inhibition [11]. Additionally both lead (Pb) and mercury (Hg) are commonly found throughout the environment and have well established detrimental effects on wildlife health. Lead and mercury are released into the environment by anthropogenic processes such as spent lead ammunition or by-products of the combustion of fossil fuels, and can lead to behavioral and neurological abnormalities.

This manuscript examines the influence of parasitic infections, organochlorine pesticides, and metal toxicity as a potential contributor to the lesser prairie-chicken decline.

## Methods

The bodies of nine hunter-harvested lesser prairie-chickens from Kansas were donated to the Wildlife Toxicology Laboratory at The Institute of Environmental and Human Health (TIEHH), Texas Tech University for extensive evaluation for the presence of eyeworms, organochlorine pesticides, and toxic metals. These lesser prairie-chickens were harvested by hunters in Kansas during a limited hunting season. Specimens were salvaged from a registered taxidermist in the taxidermy specimen preparation process. Prior to processing by the taxidermists, each eyeball and its associated lacrimal ducts and tissues were removed and put into separate 70% ethanol vials. The eye sockets were examined for any remaining eyeworms and they were placed into their respective vials. In coordination with samples supplied by registered taxidermists, we were allocated the eyes and their associated ducts and glands, femur, liver, and breast muscle.

### Eyeworm Examination

Each bird was thawed and examined for eyeworms. The examination started by removing the lacrimal duct, gland, and tissue from the eyeball and teasing them apart to look for eyeworms. Any eyeworms that were found during the examination process were placed in a physiological saline holding media. When all the examinations were complete, eyeworms were then transferred into a 70% ethanol+8% glycerin vial for preservation. Voucher parasite specimens of *O. petrowi* (USNPC No. 108249) were deposited in the U.S. National Parasite Collection, Beltsville, Maryland. Prevalence refers to the number of lesser prairie-chickens infected with *O. petrowi* in the sample divided by total number of lesser prairie-chickens examined in the sample, and mean is the number of *O. petrowi* found in the lesser prairie-chickens sampled by the total number of lesser prairie-chickens examined [12].

### Contaminant Analysis

Samples of breast muscle, liver, and fat from the lower breast were taken from all nine donated birds. These tissues were extracted using a general QuEChERS method and analyzed by GC-ECD for 20 organochlorine pesticides and metabolites: hexachlorocyclohexane (HCH; alpha, beta, gamma (lindane), and delta isomers), alpha and gamma chlordane, heptachlor and heptachlor epoxide, DDT, DDE, DDD, methoxychlor, aldrin, dieldrin, endrin, endrin aldehyde, endrin ketone, endosulfan I, endosulfan II, and endosulfan sulfate [13]. The QuEChERS method started by taking approximately 3 g wet weight tissue that was then freeze dried and added to a 50 mL centrifuge tube containing 4,000 mg anhydrous magnesium sulfate and 1,000 mg anhydrous sodium chloride (United Chemical Technologies, Bristol, PA, USA). To this tube 15–20 mL acetonitrile was added with tetrachloro-*m*-xylene as an internal standard. After vortexing and centrifugation (3,000 rpm for 10 min), the extract was decanted and transferred into a 15 mL QuEChERS cleanup centrifuge tube containing 900 mg anhydrous magnesium sulfate, 300 mg primary-secondary amine (PSA) exchange material, and 150 mg endcapped C18 (United Chemical Technologies, Bristol, PA, USA).

Samples were analyzed on a Hewlett-Packard 6890 gas chromatograph equipped with two Agilent columns (primary: DB-17 ms: 30 m×0.32 mm×0.25 µm; secondary: DB-XLB: 30 m×0.32 mm×0.50 µm) and two μECD detectors. Inlet and detector temperatures were 220°C and 300°C (both detectors), respectively. A volume of 2 µL was injected in pulsed splitless mode with a pulse pressure of 40.0 psi, pulse time of 0.20 min, purge flow of 44.8 mL/min and purge time of 1.00 min. Each standard was made by spiking chicken breast extract with a certified OC pesticide mixture (Restek, Belafonte, PA, USA) and TCMX (Accustandard, New Haven, CT, USA).

Additionally, livers and a portion of each femur were extracted from all lesser prairie-chickens and sent to Trace Analysis Inc. (Lubbock, TX, USA). Livers were analyzed for elevated mercury levels and femurs were analyzed for elevated lead levels. Livers were tested for mercury using modified EPA method SW-846 7471B. [14]. First 5 mL of DI water was added to a 0.5–0.6 g portion of well-homogenized, freeze-dried sample. This mixture was heated for 2 minutes at 95±3°C. After cooling, an additional 50 mL of DI water was added, followed by 15 mL of potassium permanganate solution, and each sample was allowed to sit for a minimum of 15 minutes. Samples were mixed thoroughly and then heated at 95±3°C for 30 minutes. After cooling, 6 mL of sodium chloride hydroxylamine sulfate solution was added to each sample. These samples were then analyzed using a CETAC M-6100 Cold Vapor Atomic Absorbance mercury analyzer. Absorbance for Hg was measured at 253.7 nm. Femur samples were prepared for Pb analysis using a slightly modified version of modified EPA method SW-846 3050B [15]. Freeze dried femurs were digested for one hour at room temperature using 5 mL of HNO_3_. An additional 5 mL of HNO_3_ was added to each sample; samples were then heated to 100°C. The heating process continued until samples were completely digested and the digestant appeared clear. Once the digestion was complete, the samples were cooled and 10 mL of 30% H_2_O_2_ was added. Samples were then heated again until foaming subsided. At this point, 10 mL of concentrated HCl was added and samples were heated until the volume was reduced to 5 mL. Samples were subsequently diluted to 50 mL using DI water. Analysis of femurs for lead followed EPA method SW-846 6010C using a Perkin Optima 8300 Duel View Inductively Coupled Plasma-Optical Emission Spectrometer. Lead was measured at a wavelength of 220.353 nm in axial mode.

Method detection limits (MDLs) ranged from 8.0 ng/g for heptachlor epoxide to 70 ng/g for methoxychlor. In general, most MDLs are approximately10 ng/g. Generally speaking, organochlorine pesticide residues in the tissues of lesser prairie-chickens were relatively low to barely detectable. Standard detection limits for lead was 0.263 µg/g and mercury's was 0.00354 µg/g.

## Results

**Figure 1:**
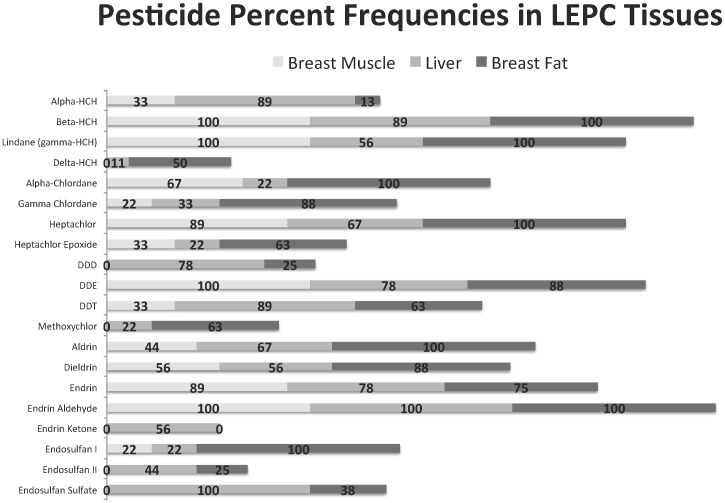
Percent frequencies of organochlorine pesticide detections, including pesticides above and below method detection limits, in lesser prairie-chickens (*Tympanuchus pallidicinctus*) by tissue type, Kansas, USA. ^*^Method detection limits for heptachlor epoxide were 8 ng/g, 70 ng/g for methoxychlor, and 10 ng/g for all other organochlorines.

Of the 9 lesser prairie-chickens examined, 7 were found to be infected with a total of 49 eyeworms. Prevalence of infection was 78% with a mean abundance of 5.44±6.02 [range:0–16]. Endrin aldehyde was detected but could not be quantitated due to partial removal in the QuEChERS cleanup step. The poor recovery of endrin aldehyde could have been a result of removal by PSA in the extraction step. Nevertheless, endrin aldehyde was detected on the primary column. Figure 1 shows the detection frequencies of each pesticide by tissue. Most residues were below method detection limits. The range and averages for pesticides above detection limits was recorded in Table 1. Even those pesticides found above detection limits are in the low ng/g range, which means it's unlikely that these levels pose a direct threat to lesser prairie-chicken health. Notable is the ubiquitous presence of endrin aldehyde across all tissues. Endrin aldehyde as well as DDE are common metabolites found in many birds including lesser prairie-chickens [16]. The presence of these organochlorines in low concentrations is consistent with the historic (and not recent) use of pesticides in the region.

**Table 1:** Average and range of select pesticides found in lesser prairie-chickens (*Tympanuchus pallidicinctus*) tissues (ng/g wet weight)*, from Kansas, USA.

Pesticide	Breast Muscle (n = 9)	Liver (n = 9)	Breast Fat (n = 8)
Alpha-HCH		14 (n = 1)	
Beta-HCH	10 (n = 1)	21 (11–32) (n = 3)	17 (n = 1)
Gamma-HCH			32 (13–64) (n = 3)
Alpha Chlordane			17 (n = 1)
Gamma Chlordane			12 (n = 1)
Heptachlor	11 (9.9–13) (n = 4)	18 (9–39) (n = 6)	43 (16–120) (n = 8)
Heptachlor Epoxide			20 (8–33) (n = 2)
DDD		35 (n = 1)	
DDE			27 (n = 1)
DDT		35 (13–74) (n = 3)	
Aldrin		42 (n = 1)	
Endrin		21 (20–21) (n = 2)	43 (n = 1)
Endrin Ketone		35 (16–57) (n = 5)	
Endosulfan I			27 (n = 1)
Endosulfan Sulfate		31 (24–38) (n = 2)	

*Values and pesticides shown are only those above detection limits. Endrin aldehyde could not be quantitated.

Note: Method detection limits for heptachlor epoxide were 8 ng/g, 70 ng/g for methoxychlor, and 10 ng/g for all other organochlorines.

Lead levels in the nine femur samples averaged 0.86 µg/g and only one of the femur samples was found to have lead above detection limits at 5.66 µg/g. This amount of lead is relatively low and not consistent with toxic lead exposure ranging between 2–8 µg/g [17], [18]. Liver samples averaged 0.04 µg/g of mercury and no samples had mercury above the 0.263 µg/g detection limit.

## Discussion

While our sample size is relatively low, it appears that lesser prairie-chickens in Kansas are not being exposed to a large number of organochlorines or two metals which are common throughout the environment. Since organochlorine pesticides have been banned or restricted, the likelihood of them exerting toxicity is being reduced daily. These pesticides are constantly being degraded in the environment and exposure is unlikely. However, this study only measured a subsample of potential organochlorine pesticides and didn't look at both organophosphate and carbamate pesticides, which means pesticides shouldn't be completely discarded as potential dangers to lesser prairie-chickens.

But, this is not the case when it comes to being exposed to potential eyeworm infection. Our eyeworm infection findings in the present study are considerably high and comparable to Robel et al. (2003) infection rates of *O. petrowi* in lesser prairie-chickens (95% infection), considering a further reduced population today.

A continuing presence of eyeworms is being reported in lesser prairie-chicken which could indicate that this parasite could potentially be a contributing factor to lesser prairie-chicken decline and have deeper implications than what was previously known. Previous research on the red grouse (*Lagopus lagopus scoticus*) in Scotland has shown that high parasite burdens can ultimately make a host more susceptible to predation [19]. Additional studies have also shown that increased parasite loads can correlate with the host population decline and reduced parasite loads throughout the populations correlate with an upward trend in the host population [20]. Eyeworm infection rates in northern bobwhites of the Rolling Plains region of Texas have been reported at greater than 90% in sampled birds and there is concern that the population of bobwhites is declining [4].

The infection rate throughout this region was much higher than what was previously thought which suggests that either the infection may have increased or was underestimated and this could be similar to what is happening in Kansas. However, because these birds are hunter shot, there may be some bias that these lesser prairie-chickens were more susceptible to being shot because of their increased parasitism than a lesser prairie-chicken that was eyeworm infection free.

The erratic behavior of lesser prairie-chickens flying into stationary objects is very similar to that of other gallinaceous or ground feeding birds, which have been found to be infected with the parasitic eyeworm *Oxyspirura petrowi* [21], [22]. Eyeworms are known blood feeding nematodes that can cause inflammation and edema behind the eyes of other gallinaceous birds. A heavy infection of eyeworm may render lesser prairie-chickens susceptible to vision problems which may impair their ability to find food or escape from predators, and a heavy infection likely causes increased energy expenditure.

Despite efforts to restore habitat and limit hunting in areas where lesser prairie-chickens are commonly found, much of the population is steadily declining to the point where they are now threatened. Parasites like the eyeworm, which we believe has been underestimated as a factor, could silently be impacting the population by compromising vision which is a necessity for predator avoidance and finding/securing food. Additionally, new conservation practices by biologists and landowners have been to add fence signs and/or colored marking tape on fences to help reduce bird/fence collisions. The idea of this practice is to enable the lesser prairie-chicken to see the fence lines; however, this conservation practice may be counterintuitive. Marking the fences with signs provides no net benefit to the lesser prairie-chickens by being there and may actually provide targets with increased probability of impact by visually impaired birds. Further research needs to be conducted on the eyeworm and the impact they have on their infected host, including lesser prairie-chickens, so better management practices can be established.
